# A Novel Virus-Patch Dynamic Model

**DOI:** 10.1371/journal.pone.0137858

**Published:** 2015-09-14

**Authors:** Lu-Xing Yang, Xiaofan Yang

**Affiliations:** 1 College of Computer Science, Chongqing University, Chongqing, China; 2 School of Software Engineering, Chongqing University, Chongqing, China; Southwest University, CHINA

## Abstract

The distributed patch dissemination strategies are a promising alternative to the conventional centralized patch dissemination strategies. This paper aims to establish a theoretical framework for evaluating the effectiveness of distributed patch dissemination mechanism. Assuming that the Internet offers P2P service for every pair of nodes on the network, a dynamic model capturing both the virus propagation mechanism and the distributed patch dissemination mechanism is proposed. This model takes into account the infected removable storage media and hence captures the interaction of patches with viruses better than the original SIPS model. Surprisingly, the proposed model exhibits much simpler dynamic properties than the original SIPS model. Specifically, our model admits only two potential (viral) equilibria and undergoes a fold bifurcation. The global stabilities of the two equilibria are determined. Consequently, the dynamical properties of the proposed model are fully understood. Furthermore, it is found that reducing the probability per unit time of disconnecting a node from the Internet benefits the containment of electronic viruses.

## 1 Introduction

Electronic viruses, ranging from host-dependent viruses and network worms to other malicious codes such as Trojans and spyware, have posed a serious threat to our daily work and life [1]. Even more serious, the highly popularized networks, ranging from the Internet and the world wide web to various social networks, offer the major channel for the fast spread of electronic viruses. Consequently, the issue of how to suppress the rampancy of electronic infections on networks has long received considerable attention from the network security community.

The patches for viruses are recognized as the major means of detecting and clearing viruses resident at individual network nodes. For the patches to play a full role, new patches must be disseminated to all nodes on the network in a remarkably short period of time. There are two fundamentally different kinds of patch dissemination strategies: the *centralized* strategies, in which a central node disseminates new patches directly to all other nodes in the network, and the *distributed* strategies, in which every newly patched node forwards the patches to some or all of its neighbors according to a well-designed protocol [2–4]. Due to the limited bandwidth of the Internet, the time needed by performing a centralized patch dissemination strategy is often unacceptably long. The distributed patch dissemination strategies are regarded as a promising alternative to their centralized analogs, because the negative impact of the limited bandwidth on the patch dissemination can be reduced significantly.

The design of good patch dissemination strategies is closely related to the evaluation of effectiveness of different patch dissemination strategies. One feasible approach to the evaluation of a patch dissemination strategy is to establish a compartment-based dynamic model capturing both the virus propagation mechanism and the patch dissemination strategy, and then to determine the trend of the number or proportion of infected nodes in the network by analyzing the dynamical properties of the model; a patch dissemination strategy is regarded as *effective* or *ineffective* depending on whether or not the proportion of infected nodes approaches an acceptably low value. Kephart and White’s seminal work in the early 1990s opened the door to the compartment modeling of computer infections [5]. From then on, a multitude of epidemic models for electronic viruses, ranging from ordinary models [6–12] and delayed models [13–16] to impulsive models [17–20], have been proposed. All these models capture the centralized patch dissemination mechanism. As a result, they are not suited to the situations of distributed patch dissemination.

Recently, Zhu et al. [21] proposed an epidemic model for electronic viruses, which is known as the *original SIPS model* in this paper. To a certain extent, this model captures the distributed patch dissemination mechanism, because every recently patched node is assumed to have a chance to forward the patches to a neighboring node. Consequently, this model offers a good start point for assessing the effectiveness of different distributed patch dissemination strategies. The model exhibits complex dynamical properties. Specifically, the model admits up to four potential equilibria, among which two are virus-free and the other two are virulent, and each of the four equilibria can be globally stable under proper conditions. As a result, the viruses on the network may die out or persist depending on the relationship among the model-related parameters.

Apart from the Internet as a channel for virus spreading, various removable storage media, including flash disks and portable hard disks, offer the second channel for virus propagation. The original SIPS model, however, ignores the existence of infected removable storage media. To accurately evaluate the effectiveness of the distributed patch dissemination mechanism, a virus-patch mixed model that takes into account infected removable storage media should be introduced.

This paper is intended to introduce a theoretical framework for evaluating the effectiveness of distributed patch dissemination mechanism. Assuming that the Internet offers P2P service for every pair of nodes on the network, a virus-patch dynamic model incorporating the impact of infected removable storage media is suggested. Certainly, this model captures the interaction of patches with viruses better than the original SIPS model. Surprisingly, our model exhibits much simpler dynamic properties than the original SIPS model. Specifically, our model admits only two potential (viral) equilibria and undergoes a fold bifurcation. The global stabilities of the two equilibria are determined. Consequently, the dynamical properties of the proposed model are fully understand. Furthermore, it is found that reducing the probability per unit time of disconnecting a node from the Internet helps suppress electronic viruses.

The remainder of this paper is organized in this fashion: Section 2 describes the new model. Section 3 computes the two potential equilibria for the model. Sections 4 and 5 are devoted to examining the local and global stabilities of the equilibria, respectively. Several numerical examples are given in Section 6. Finally, Section 7 summarizes this work and points out some future topics of research.

## 2 Model formulation

This section aims to introduce the new virus-patch dynamic model. For brevity, smart electronic devices are referred to as *nodes*. It is assumed that the Internet offers P2P service for every pair of nodes on the network. Due to the limited carrying capacity of the Internet, it is assumed that the number of nodes on the network, denoted *N*, is unvaried over time.

Every node is assumed to be in one of three possible states: *susceptible*, *infected*, and *patched*. Susceptible nodes are not installed with the newest patch and hence have no immunity to new viruses, whereas patched nodes are installed with the newest patch and hence possess temporary immunity to new viruses. Let *S*(*t*), *I*(*t*), and *P*(*t*) denote the average numbers of susceptible, infected, and immune nodes on the network at time *t*, respectively. Clearly, *S*(*t*) + *I*(*t*) + *P*(*t*) ≡ *N*. For the modeling purpose, the following hypotheses are imposed.

(H1) The nodes outside the network are all susceptible.
(H2) The nodes outside the network are connected to the network at constant rate *μ* > 0.
(H3) Every node in the network is disconnected from the network with constant probability per unit time *δ* > 0. Clearly, we have $\frac{\mu }{\delta }=N$ .
(H4) Due to connections with infected nodes, at time *t* every susceptible node in the network gets infected with probability per unit time *β*_1_ *I*(*t*), where *β*_1_ > 0 is a constant. This hypothesis captures the distributed nature of virus propagation.
(H5) Due to existence of infected removable storage media, every susceptible node in the network gets infected with constant probability per unit time *β*_2_ > 0.
(H6) Due to connections with patched nodes, at time *t* every susceptible or infected node in the network acquires the newest patch with probability per unit time *γ*_1_ *P*(*t*), where *γ*_1_ > 0 is a constant. This hypothesis captures the distributed nature of patch dissemination.
(H7) Due to system reinstallation, every infected node in the internet becomes susceptible with constant probability per unit time *γ*_2_ > 0.
(H8) Due to patch invalidation, every patched node in the network becomes susceptible with constant probability per unit time *α* > 0.

**Fig 1:**
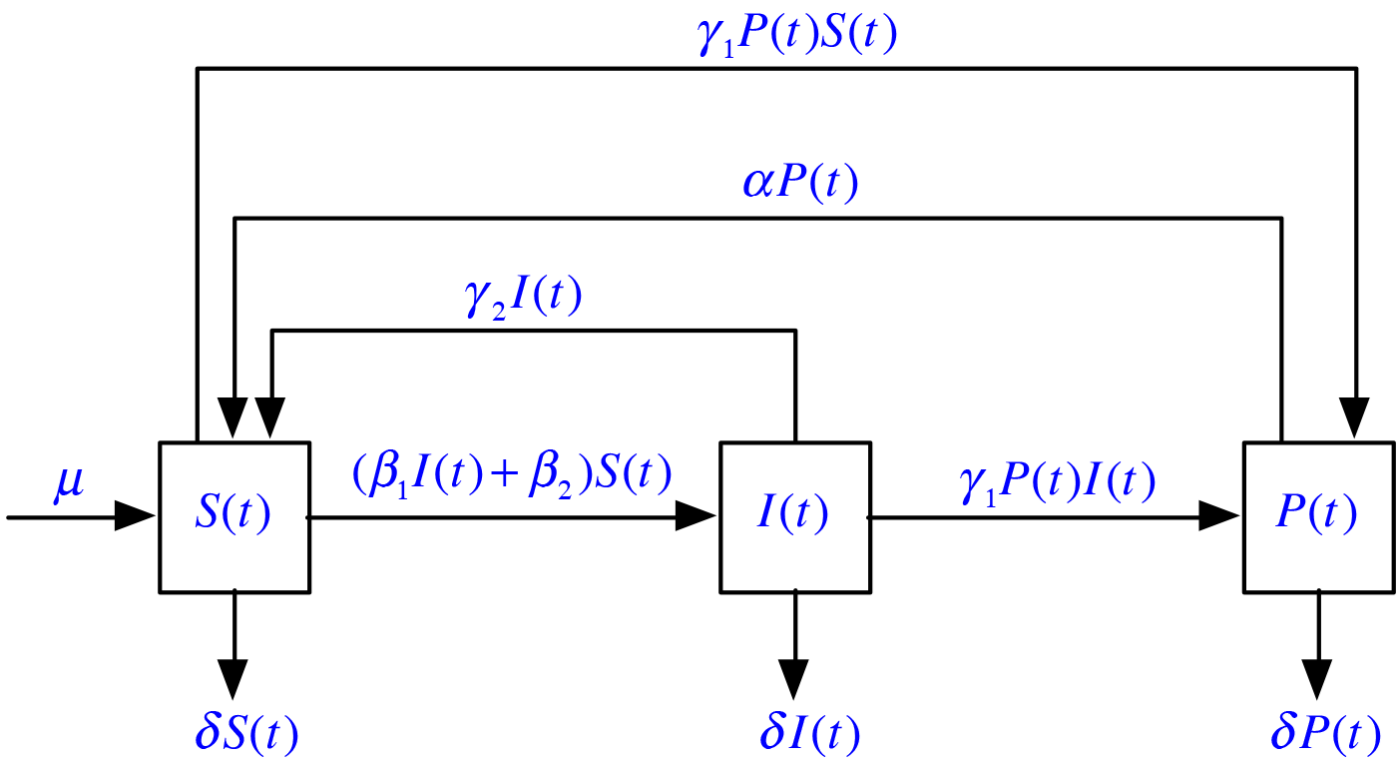
The diagram for the new model.

This collection of hypotheses can be presented in the form of Fig 1. On this basis, our new model can be formulated as the following differential dynamical system:

$$\begin{array}{c} \left\{ \begin{array}{c} \frac{dS(t)}{dt}=\mu -\beta_{1}S\left(t\right)I\left(t\right)-\beta_{2}S\left(t\right)-\gamma_{1}S\left(t\right)P\left(t\right)+\gamma_{2}I\left(t\right)+\alpha P\left(t\right)-\delta S\left(t\right),\\ \frac{dI(t)}{dt}=\beta_{1}S\left(t\right)I\left(t\right)+\beta_{2}S\left(t\right)-\gamma_{1}I\left(t\right)P\left(t\right)-\gamma_{2}I\left(t\right)-\delta I\left(t\right),\\ \frac{dP(t)}{dt}=\gamma_{1}S\left(t\right)P\left(t\right)+\gamma_{1}I\left(t\right)P\left(t\right)-\alpha P\left(t\right)-\delta P\left(t\right), \end{array}\right. \end{array}$$ (1)

with initial condition (*S*(0), *I*(0), *P*(0)) ∈ Ω, where

$$\begin{array}{c} \Omega =\{\left(S,I,P\right)\in R_{+}^{3}:S+I+P=\frac{\mu }{\delta }\}. \end{array}$$

It is easily verified that Ω is positively invariant for the system.

As $S(t)+I(t)+P(t)\equiv \frac{\mu }{\delta }$ , system (1) reduces to the following two-dimensional dynamical system:

$$\begin{array}{c} \left\{ \begin{array}{c} \frac{dI(t)}{dt}=\frac{\beta_{2}\mu }{\delta }+\left(\frac{\beta_{1}\mu }{\delta }-\beta_{2}-\gamma_{2}-\delta \right)I\left(t\right)-\beta_{2}P\left(t\right)-\beta_{1}I^{2}\left(t\right)\\ \;\;\;-\left(\beta_{1}+\gamma_{1}\right)I\left(t\right)P\left(t\right),\\ \frac{dP(t)}{dt}=\left(\frac{\gamma_{1}\mu }{\delta }-\alpha -\delta \right)P\left(t\right)-\gamma_{1}P^{2}\left(t\right), \end{array}\right. \end{array}$$ (2)

with initial condition (*I*(0), *P*(0)) ∈ Ω*, where

$$\begin{array}{c} \Omega^{*}=\left\{\left(I,P\right)\in R_{+}^{2}:I+P\leq \frac{\mu }{\delta }\right\}. \end{array}$$

It is easily verified that Ω* is positively invariant for the system.

One of our major tasks is to determine the trend of *I*(*t*) by studying model (2).

## 3 Equilibria

An *equilibrium* for a differential dynamical system is a state of the system that is unvaried over time. The first step to understanding a differential dynamical system is to figure out what equilibria it admits.

Now, let us pick out all equilibria of system (1). Let ${\tilde{\text{E}}}_{1}=(S_{1}^{*},I_{1}^{*},P_{1}^{*})$ , ${\tilde{\text{E}}}_{2}=(S_{2}^{*},I_{2}^{*},P_{2}^{*})$ , where $P_{1}^{*}=0$ , $P_{2}^{*}=\frac{\mu }{\delta }-\frac{\alpha +\delta }{\gamma_{1}}$ ,

$$\begin{array}{c} S_{1}^{*}=\frac{\mu }{2\delta }-\frac{1}{2\beta_{1}}\sqrt{{\left(\beta_{2}+\gamma_{2}+\delta -\frac{\beta_{1}\mu }{\delta }\right)}^{2}+\frac{4\beta_{1}\beta_{2}\mu }{\delta }}+\frac{1}{2\beta_{1}}\left(\beta_{2}+\gamma_{2}+\delta \right), \end{array}$$

$$\begin{array}{c} I_{1}^{*}=\frac{\mu }{2\delta }+\frac{1}{2\beta_{1}}\sqrt{{\left(\beta_{2}+\gamma_{2}+\delta -\frac{\beta_{1}\mu }{\delta }\right)}^{2}+\frac{4\beta_{1}\beta_{2}\mu }{\delta }}-\frac{1}{2\beta_{1}}\left(\beta_{2}+\gamma_{2}+\delta \right), \end{array}$$

$$\begin{array}{ccc} S_{2}^{*} & = & \frac{\alpha +\delta }{2\gamma_{1}}-\frac{1}{2\beta_{1}}\sqrt{{\left(\beta_{2}+\gamma_{2}+\frac{\gamma_{1}\mu }{\delta }-\alpha -\frac{\beta_{1}\left(\alpha +\delta \right)}{\gamma_{1}}\right)}^{2}+\frac{4\beta_{1}\beta_{2}\left(\alpha +\delta \right)}{\gamma_{1}}}\\ & & +\frac{1}{2\beta_{1}}\left(\beta_{2}+\gamma_{2}+\frac{\gamma_{1}\mu }{\delta }-\alpha -\frac{\beta_{1}\left(\alpha +\delta \right)}{\gamma_{1}}\right), \end{array}$$

and

$$\begin{array}{ccc} I_{2}^{*} & = & \frac{\alpha +\delta }{2\gamma_{1}}+\frac{1}{2\beta_{1}}\sqrt{{\left(\beta_{2}+\gamma_{2}+\frac{\gamma_{1}\mu }{\delta }-\alpha -\frac{\beta_{1}\left(\alpha +\delta \right)}{\gamma_{1}}\right)}^{2}+\frac{4\beta_{1}\beta_{2}\left(\alpha +\delta \right)}{\gamma_{1}}}\\ & & -\frac{1}{2\beta_{1}}\left(\beta_{2}+\gamma_{2}+\frac{\gamma_{1}\mu }{\delta }-\alpha -\frac{\beta_{1}\left(\alpha +\delta \right)}{\gamma_{1}}\right). \end{array}$$

**Theorem 3.1**. *Consider system (1)*.

- *There is a unique equilibrium*, ${\tilde{\text{E}}}_{1}$ , *if* $\frac{\mu }{\delta }\leq \frac{\alpha +\delta }{\gamma_{1}}$ .
- *There are exactly two equilibria*, ${\tilde{\text{E}}}_{1}$ *and* ${\tilde{\text{E}}}_{2}$ , if $\frac{\mu }{\delta }>\frac{\alpha +\delta }{\gamma_{1}}$ .

**Proof**. *Any equilibrium for system (1) must be a solution to the bilinear algebraic system*

$$\begin{array}{c} \left\{ \begin{array}{ccc} \mu -\beta_{1}SI-\beta_{2}S-\gamma_{1}SP+\gamma_{2}I+\alpha P-\delta S & = & 0,\\ \beta_{1}IS+\beta_{2}S-\gamma_{1}IP-\gamma_{2}I-\delta I & = & 0,\\ \gamma_{1}SP+\gamma_{1}IP-\alpha P-\delta P & = & 0. \end{array}\right. \end{array}$$ (3)

*Direct calculations show that system (1) has at most one equilibrium*, ${\tilde{\text{E}}}_{1}$ , *if* *P* = 0, *and system (1) has at most two equilibria*, ${\tilde{\text{E}}}_{1}$ *and* ${\tilde{\text{E}}}_{2}$ , *if* *P* ≠ 0.

*First, suppose* *P* = 0. *Canceling S from the first two equations of system (3), and rearranging the terms, we get that* $I_{1}^{*}$ *is a positive root of the quadratic equation*

$$\begin{array}{c} f\left(I\right)=\beta_{1}I^{2}+\left(\beta_{2}+\gamma_{2}+\delta -\frac{\beta_{1}\mu }{\delta }\right)I-\frac{\beta_{2}\mu }{\delta }=0. \end{array}$$ (4)

*As* $f(0)=-\frac{\beta_{2}\mu }{\delta }<0$ *and* $f(\frac{\mu }{\delta })=\frac{(\gamma_{2}+\delta )\mu }{\delta }>0$ , *it follows that, in any case*, ${\tilde{\text{E}}}_{1}$ *is an equilibrium*.

*Clearly*, ${\tilde{\text{E}}}_{2}$ *is an equilibrium only if* $\frac{\mu }{\delta }>\frac{\alpha +\delta }{\gamma_{1}}$ . *Now, suppose* *P* ≠ 0. *Canceling S and P from system (3) and rearranging the terms, we get that* $I_{2}^{*}$ *is a positive root of the quadratic equation*

$$\begin{array}{c} g\left(I\right)=\beta_{1}I^{2}+\left(\beta_{2}+\gamma_{2}+\frac{\gamma_{1}\mu }{\delta }-\alpha -\frac{\beta_{1}\left(\alpha +\delta \right)}{\gamma_{1}}\right)I-\frac{\beta_{2}\left(\alpha +\delta \right)}{\gamma_{1}}=0. \end{array}$$ (5)

*As* $g(0)=-\frac{\beta_{2}(\alpha +\delta )}{\gamma_{1}}<0$ , *it follows that* ${\tilde{\text{E}}}_{2}$ *is an equilibrium if* $\frac{\mu }{\delta }>\frac{\alpha +\delta }{\gamma_{1}}$ *and*

$$\begin{array}{c} g\left(\frac{\alpha +\delta }{\gamma_{1}}\right)=\left(\gamma_{2}+\frac{\gamma_{1}\mu }{\delta }-\alpha \right)\frac{\alpha +\delta }{\gamma_{1}}>0. \end{array}$$

*As* $\frac{\mu }{\delta }>\frac{\alpha +\delta }{\gamma_{1}}$ *implies* $g(\frac{\alpha +\delta }{\gamma_{1}})>0$ , *it follows that* ${\tilde{\text{E}}}_{2}$ *is indeed an equilibrium if* $\frac{\mu }{\delta }>\frac{\alpha +\delta }{\gamma_{1}}$ . *The proof is complete*.

An equilibrium for system (1) or (2) is *virus-free* if its I-component is zero, otherwise the equilibrium is *viral*. It is easily verified that (a) ${\tilde{\text{E}}}_{1}$ is viral, and (b) ${\tilde{\text{E}}}_{2}$ is viral if $\frac{\mu }{\delta }>\frac{\alpha +\delta }{\gamma_{1}}$ . As a result, system (1) possesses no virus-free equilibrium.

Let ${\text{E}}_{1}^{*}=(I_{1}^{*},P_{1}^{*})$ , ${\text{E}}_{2}^{*}=(I_{2}^{*},P_{2}^{*})$ . As a consequence of Theorem 3.1, we have the following result.

**Theorem 3.2**. *Consider system (2)*.

- *There is a unique equilibrium*, ${\text{E}}_{1}^{*}$ , if $\frac{\mu }{\delta }\leq \frac{\alpha +\delta }{\gamma_{1}}$ .
- *There are exactly two equilibria*, ${\text{E}}_{1}^{*}$ *and* ${\text{E}}_{2}^{*}$ , *if* $\frac{\mu }{\delta }>\frac{\alpha +\delta }{\gamma_{1}}$ .

Clearly, ${\text{E}}_{1}^{*}$ is viral, and ${\text{E}}_{2}^{*}$ is viral if $\frac{\mu }{\delta }>\frac{\alpha +\delta }{\gamma_{1}}$ . As a result, system (2) admits no virus-free equilibrium. This theorem clearly shows that under model (2), it is impossible to eradicate viruses on the network once for all.

## 4 Local stability analysis

Given an equilibrium E* for a differential dynamical system. E* is *stable* if any orbit for the system that starts from a point near E* always stays in the proximity of E*, otherwise E* is a *repeller*. E* is *attracting* if any orbit for the system that starts from a point near E* approaches E*. E* is *asymptotically stable* if it is stable and attracting.

Given an equilibrium for a differential dynamic system, the next thing to do is to figure out its local stability. In this section, the local stabilities of the two equilibria, ${\text{E}}_{1}^{*}$ and ${\text{E}}_{2}^{*}$ , for system (2) are examined.

**Theorem 4.1**. *Consider system (1)*.

-
${\tilde{\text{E}}}_{1}$ *is asymptotically stable if* $\frac{\mu }{\delta }<\frac{\alpha +\delta }{\gamma_{1}}$ .
-
${\tilde{\text{E}}}_{1}$ *is a saddle point, with one positive eigenvalue and two negative eigenvalues, if* $\frac{\mu }{\delta }>\frac{\alpha +\delta }{\gamma_{1}}$ .

**Proof**. *The Jacobian of system (1) evaluated at* ${\tilde{\text{E}}}_{1}$ *is*

$$\begin{array}{ccc} & & (\begin{array}{ccc} -\beta_{1}I_{1}^{*}-\beta_{2}-\delta & \gamma_{2}-\beta_{1}S_{1}^{*} & \alpha -\gamma_{1}S_{1}^{*}\\ \beta_{1}I_{1}^{*}+\beta_{2} & \beta_{1}S_{1}^{*}-\gamma_{2}-\delta & -\gamma_{1}I_{1}^{*}\\ 0 & 0 & \frac{\gamma_{1}\mu }{\delta }-\alpha -\delta \end{array}). \end{array}$$

*The associated characteristic equation is*

$$\begin{array}{c} \left(\xi +\delta \right)\left(\xi -\frac{\gamma_{1}\mu }{\delta }+\alpha +\delta \right)\left(\xi +\beta_{2}+\delta +\gamma_{2}+\beta_{1}I_{1}^{*}-\beta_{1}S_{1}^{*}\right)=0. \end{array}$$

*Note from the second equation of algebraic system (3) that* $\beta_{1}S_{1}^{*}<\gamma_{2}+\delta$ , *the three roots of this equation are*

$$\begin{array}{ccc} \xi_{1} & = & -\delta <0,\\ \xi_{2} & = & \gamma_{1}\left(\frac{\mu }{\delta }-\frac{\alpha +\delta }{\gamma_{1}}\right),\\ \xi_{3} & = & \beta_{1}S_{1}^{*}-\beta_{1}I_{1}^{*}-\beta_{2}-\delta -\gamma_{2}<-\beta_{1}I_{1}^{*}-\beta_{2}<0. \end{array}$$

*So*, *ξ*_2_ < 0 *if* $\frac{\mu }{\delta }<\frac{\alpha +\delta }{\gamma_{1}}$ , *and* *ξ*_2_ > 0 *if* $\frac{\mu }{\delta }>\frac{\alpha +\delta }{\gamma_{1}}$ . *Thus, the claimed result follows from the Lyapunov theorem* [22].

**Theorem 4.2**. *Consider system (1)*. ${\tilde{\text{E}}}_{2}$ *is locally asymptotically stable if* $\frac{\mu }{\delta }>\frac{\alpha +\delta }{\gamma_{1}}$ .

**Proof**. *The Jacobian of system (1) evaluated at* *E*_2_ *is*

$$\begin{array}{ccc} & & (\begin{array}{ccc} -\beta_{1}I_{2}^{*}-\gamma_{1}P_{2}^{*}-\beta_{2}-\delta & \gamma_{2}-\beta_{1}S_{2}^{*} & \alpha -\gamma_{1}S_{2}^{*}\\ \beta_{1}I_{2}^{*}+\beta_{2} & \beta_{1}S_{2}^{*}-\gamma_{1}P_{2}^{*}-\gamma_{2}-\delta & -\gamma_{1}I_{2}^{*}\\ \gamma_{1}P_{2}^{*} & \gamma_{1}P_{2}^{*} & 0 \end{array}). \end{array}$$

*The corresponding characteristic equation is*

$$\begin{array}{c} \left(\xi +\delta \right)\left(\xi +\frac{\gamma_{1}\mu }{\delta }-\alpha -\delta \right)\left(\xi -\beta_{1}S_{2}^{*}+\beta_{1}I_{2}^{*}+\frac{\gamma_{1}\mu }{\delta }-\alpha +\beta_{2}+\gamma_{2}\right)=0. \end{array}$$

*Note that* *β*_1_ *S*_2_ < *γ*_2_+*δ*, *the three roots of this equation are*

$$\begin{array}{ccc} \xi_{1} & = & -\delta <0,\\ \xi_{2} & = & -\gamma_{1}\left(\frac{\mu }{\delta }-\frac{\alpha +\delta }{\gamma_{1}}\right)<0,\\ \xi_{3} & = & \beta_{1}S_{2}^{*}-\beta_{1}I_{2}^{*}-\frac{\gamma_{1}\mu }{\delta }+\alpha -\beta_{2}-\gamma_{2}\\ & < & \beta_{1}S_{2}^{*}-\beta_{1}I_{2}^{*}-\delta -\beta_{2}-\gamma_{2}\\ & < & -\beta_{1}I_{2}^{*}-\beta_{2}<0. \end{array}$$

*The claimed result follows from the Lyapunov theorem* [22].

**Fig 2:**
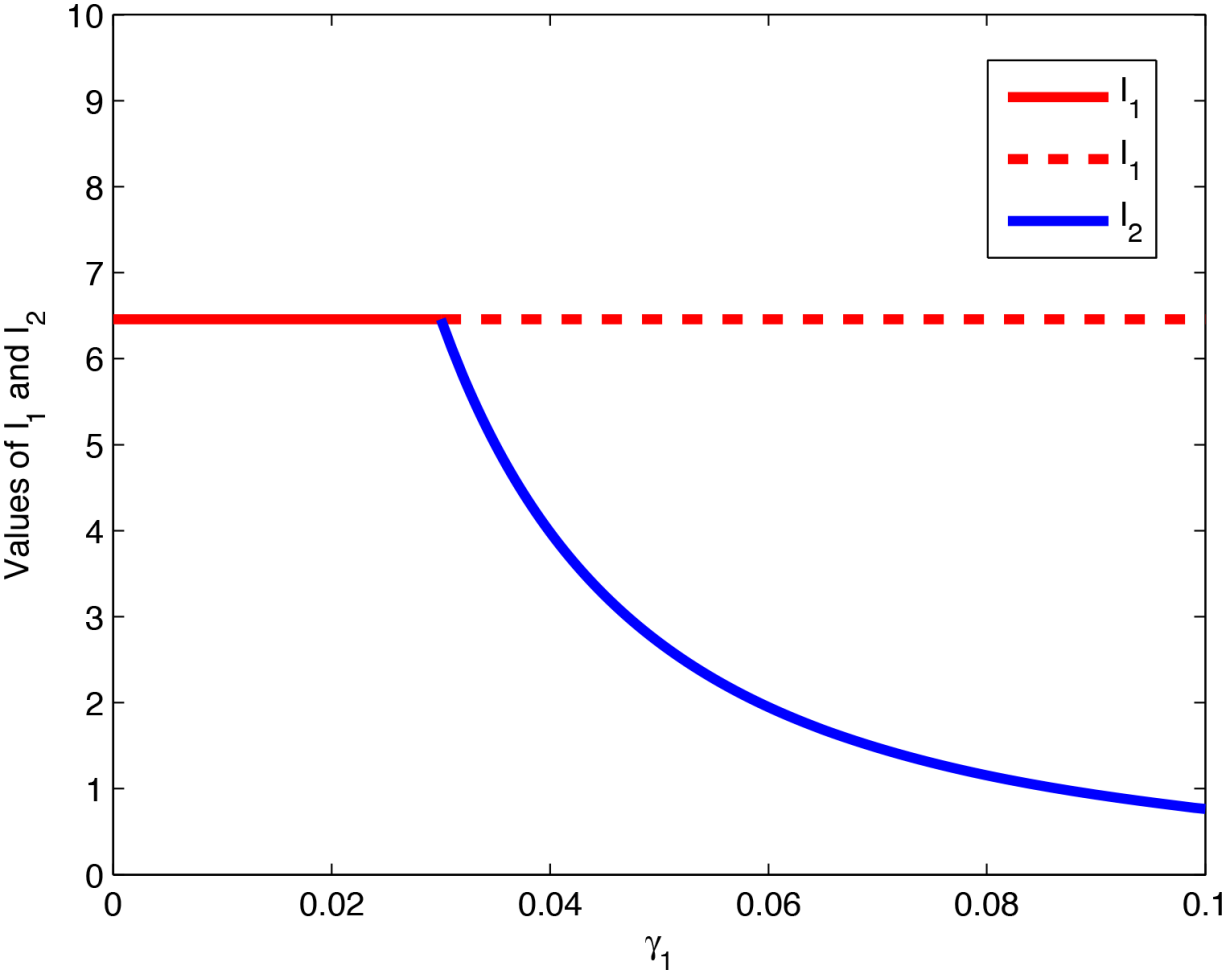
The way *I*_1_ and *I*_2_ vary with the increase of *γ*_1_.

**Remark 1**. *Theorems 4.1 and 4.2 tell us that system (1) undergoes a fold bifurcation* [23]. Fig 2 *demonstrates the way* $I_{1}^{*}$ *and* $I_{2}^{*}$ *vary with the increase of* *γ*_1_, *showing that a fold bifurcation occurs*.

As a direct consequence of Theorems 4.1 and 4.2, we have

**Theorem 4.3**. *Consider system (2)*.

-
${\text{E}}_{1}^{*}$ *is asymptotically stable if* $\frac{\mu }{\delta }<\frac{\alpha +\delta }{\gamma_{1}}$ .
-
${\text{E}}_{2}^{*}$ *is asymptotically stable and* ${\text{E}}_{1}^{*}$ *is a saddle point if* $\frac{\mu }{\delta }>\frac{\alpha +\delta }{\gamma_{1}}$ .

## 5 Global stability analysis

Given an equilibrium E* for a differential dynamical system and a subset *D* of the domain for the system containing E*. E* is *asymptotically stable with respect to D* if (a) E* is stable, and (b) any orbit starting from within *D* approaches E*.

Given the local stability of an equilibrium for a differential dynamical system, the next thing to do is to figure out its global stability. This section is devoted to examining the global stabilities of the two equilibria, **E**_1_ and **E**_2_, for system (2). For that purpose, let us briefly survey the theory of asymptotically autonomous systems.

**Definition 5.1**. *Consider a pair of n-dimensional differential dynamical systems*,

$$\begin{array}{c} \;\dot{x}=f\left(t,x\right) \end{array}$$ (6)

*and*

$$\begin{array}{c} \;\dot{x}=g\left(x\right), \end{array}$$ (7)

*defined in some positively invariant set* X ⊂ R^*n*^. *System (6) is called* asymptotically autonomous, *with system (7) as its* limit system, *if* lim_*t* → ∞_ **f**(*t*,**x**) = **g**(**x**) *holds locally uniformly in* X.

**Definition 5.2**. *The ω-limit set of a forward bounded solution* **x**(*t*) *to system (6) satisfying* **x**(*t*_0_) = **x**_0_, *denoted* *ω*(*t*_0_,**x**_0_), *is defined as* **y** ∈ *ω*(*t*_0_,**x**_0_) ⇔ **y** = lim_*j* → ∞_ **x**(*t*_*j*_) *for some sequence* *t*_*j*_ → ∞.

Below is the well-known Thieme’s Theorem concerning asymptotically autonomous systems [24].

**Theorem 5.1** (Thieme). *Let* *n* = 2 and *ω* *be the* *ω*–*limit* *set of a forward bounded solution* **x**(*t*) *of the asymptotically autonomous system (6). Assume that there exists a neighborhood of* *ω* *which contains at most finitely many equilibria of system (7). Then the following trichotomy holds*:

- *ω consists of a single equilibrium for system (7)*.
- *ω is the union of periodic orbits for system (6) and possibly of centers for system (7) that are surrounded by periodic orbits for system (6) lying in ω*.
- *ω contains equilibria for system (7) that are cyclically chained to each other in ω by orbits of system (7)*.

We are ready to make clear the global stabilities of **E**_1_ and **E**_2_. First, we have the following result.

**Lemma 5.1**. *Consider system (2)*.

- *There is no periodic solution within* Ω*.
-
${\text{E}}_{1}^{*}$ *is asymptotically stable with respect to* Ω* *if* $\frac{\mu }{\delta }<\frac{\alpha +\delta }{\gamma_{1}}$ .
-
${\text{E}}_{1}^{*}$ *is attracting with respect to* {(*I*, *P*) ∈ Ω*:*P* = 0} *and* ${\text{E}}_{2}^{*}$ *is attracting with respect to* {(*I*, *P*) ∈ Ω*:*P* ≠ 0} *if* $\frac{\mu }{\delta }>\frac{\alpha +\delta }{\gamma_{1}}$ .

**Proof**.

- *Let*
$$\begin{array}{ccc} f_{1}\left(I,P\right) & = & \frac{\beta_{2}\mu }{\delta }+\left(\frac{\beta_{1}\mu }{\delta }-\beta_{2}-\gamma_{2}-\delta \right)I-\beta_{2}P-\beta_{1}I^{2}-\left(\beta_{1}+\gamma_{1}\right)IP,\\ f_{2}\left(I,P\right) & = & \left(\frac{\gamma_{1}\mu }{\delta }-\alpha -\delta \right)P-\gamma_{1}P^{2},\\ D(I,P) & = & \frac{1}{IP}. \end{array}$$
  *In the interior of* Ω*, *we have*
$$\begin{array}{c} \frac{\partial (Df_{1})}{\partial I}+\frac{\partial (Df_{2})}{\partial P}=-\frac{\beta_{1}}{P}-\frac{\gamma_{1}}{I}-\frac{\beta_{2}}{I^{2}P}\left(\frac{\mu }{\delta }-P\right)<0. \end{array}$$
  *By the Bendixson-Dulac criterion* [22], *system (2) has no periodic orbit in the interior of* Ω*. *Now, consider an arbitrary point*, $\left(\bar{I},\bar{P}\right)$ , *on the boundary of* Ω*. *There are three possibilities, which are treated respectively*.
  -
$0\leq \bar{I}\leq \frac{\mu }{\delta }$ , $\bar{P}=0$ . *Then*, $\frac{dP(t)}{dt}{|}_{\left(\bar{I},\bar{P}\right)}=0$ .
  -
$0<\bar{P}<\frac{\mu }{\delta }$ , $\bar{I}=0$ . *Then*, $\frac{dI(t)}{dt}{|}_{\left(\bar{I},\bar{P}\right)}=\beta_{2}(\frac{\mu }{\delta }-\bar{P})>0$ .
  -
$\bar{I}+\bar{P}=\frac{\mu }{\delta }$ . *Then*, $\frac{d(I(t)+P(t))}{dt}{|}_{\left(\bar{I},\bar{P}\right)}=-\gamma_{2}\bar{I}-\alpha \bar{P}-\delta (\bar{I}+\bar{P})<0$ .
  *In view of the orbit smoothness and that there is no periodic orbit falling in the set* {(*I*, *P*) ∈ Ω*:*P* = 0}, *system (2) admits no periodic orbit in the whole* Ω*.
- *The claimed result follows from the generalized Poincare-Bendixson theorem* [22], *the first assertion of this lemma, and Corollary 2*.
- *Consider an arbitrary solution*, (*I*(*t*), *P*(*t*)), *to system (2). There are three possibilities*.
  - *P*(0) = 0. *This implies that* *P*(*t*) ≡ 0. *Plugging it into the first equation of system (2) and solving the resulting equation, we get that* $I(t)\to I_{1}^{*}$ .
  -
$P(0)=\frac{\mu }{\delta }-\frac{\alpha +\delta }{\gamma_{1}}$ . *This implies that* $P(t)\equiv \frac{\mu }{\delta }-\frac{\alpha +\delta }{\gamma_{1}}$ . *Plugging it into the first equation of system (2) and solving the resulting equation, we get that* $I(t)\to I_{2}^{*}$ .
  - *P*(0) ≠ 0, $P(0)\neq \frac{\mu }{\delta }-\frac{\alpha +\delta }{\gamma_{1}}$ . *Solving the second equation of system (2), we get*
$$\begin{array}{c} P\left(t\right)=h_{1}\left(t\right):=\frac{P_{2}^{*}}{1+\left(\frac{P_{2}^{*}}{P(0)}-1\right)e^{-\frac{\gamma_{1}}{P_{2}^{*}}t}}. \end{array}$$ (8)
    *Note that*
$$\begin{array}{c} \frac{dP(t)}{dt}=\frac{dh_{1}\left(t\right)}{dt}=h_{2}\left(t\right):=\frac{\gamma_{1}\left(\frac{P_{2}^{*}}{P(0)}-1\right)e^{-\frac{\gamma_{1}}{P_{2}^{*}}t}}{{\left[1+\left(\frac{P_{2}^{*}}{P(0)}-1\right)e^{-\frac{\gamma_{1}}{P_{2}^{*}}t}\right]}^{2}}. \end{array}$$ (9)
    *It follows from* Eqs (8) *and* (9) *that system (2) is equivalent to the following nonautonomous system*:
$$\begin{array}{c} \left\{ \begin{array}{c} \frac{dI(t)}{dt}=\frac{\beta_{2}\mu }{\delta }+\left(\frac{\beta_{1}\mu }{\delta }-\beta_{2}-\gamma_{2}-\delta \right)I\left(t\right)-\beta_{2}h_{1}\left(t\right)\\ \;\;\;-\beta_{1}I^{2}\left(t\right)-\left(\beta_{1}+\gamma_{1}\right)h_{1}^{2}\left(t\right)I\left(t\right),\\ \frac{dP(t)}{dt}=h_{2}\left(t\right), \end{array}\right. \end{array}$$ (10)
    *which converges locally uniformly to the limit system*
$$\begin{array}{c} \left\{ \begin{array}{c} \frac{dI(t)}{dt}=\frac{\beta_{2}\mu }{\delta }+\left(\frac{\beta_{1}\mu }{\delta }-\beta_{2}-\gamma_{2}-\delta \right)I\left(t\right)-\beta_{2}P_{2}^{*}\\ \;\;\;-\beta_{1}I^{2}-\left(\beta_{1}+\gamma_{1}\right){\left(P_{2}^{*}\right)}^{2}I\left(t\right),\\ \frac{dP(t)}{dt}=0. \end{array}\right. \end{array}$$ (11)
    *Clearly, system (11) has* ${\text{E}}_{2}^{*}$ *as its unique equilibrium. It follows by the Thieme’s theorem, the first assertion of this lemma, and Corollary 2 that* $I(t)\to I_{2}^{*}$ .
  *The proof is complete*.

We are ready to establish the main results in this paper.

**Theorem 5.2**. *Consider system (1)*,

-
${\tilde{\text{E}}}_{1}$ *is asymptotically stable with respect to* Ω *if* $\frac{\mu }{\delta }<\frac{\alpha +\delta }{\gamma_{1}}$ .
-
${\tilde{\text{E}}}_{1}$ *and* ${\tilde{\text{E}}}_{2}$ *are asymptotically stable with respect to* {(*S*, *I*, *P*) ∈ Ω:*P* = 0} *and* {(*S*, *I*, *P*) ∈ Ω:*P* ≠ 0}, *respectively, if* $\frac{\mu }{\delta }>\frac{\alpha +\delta }{\gamma_{1}}$ .

**Proof**. *The claims follow by combining Theorems 4.1–4.2 with Lemma 5.1*.

This theorem presents the complete dynamics for system (1). It is concluded from this theorem that (a) the number of infected nodes approaches $I_{1}^{*}$ if $\frac{\mu }{\delta }<\frac{\alpha +\delta }{\gamma_{1}}$ , (b) the number of infected nodes approaches $I_{1}^{*}$ if $\frac{\mu }{\delta }>\frac{\alpha +\delta }{\gamma_{1}}$ and initially there is no patched node, and (c) the number of infected nodes approaches $I_{2}^{*}$ if $\frac{\mu }{\delta }>\frac{\alpha +\delta }{\gamma_{1}}$ and initially there exist patched nodes. Consequently, the trend of the number of infected nodes is already perfectly clear.

## 6 Numerical examples and discussions

First, let us illustrate the function of Theorem 5.2.

**Fig 3:**
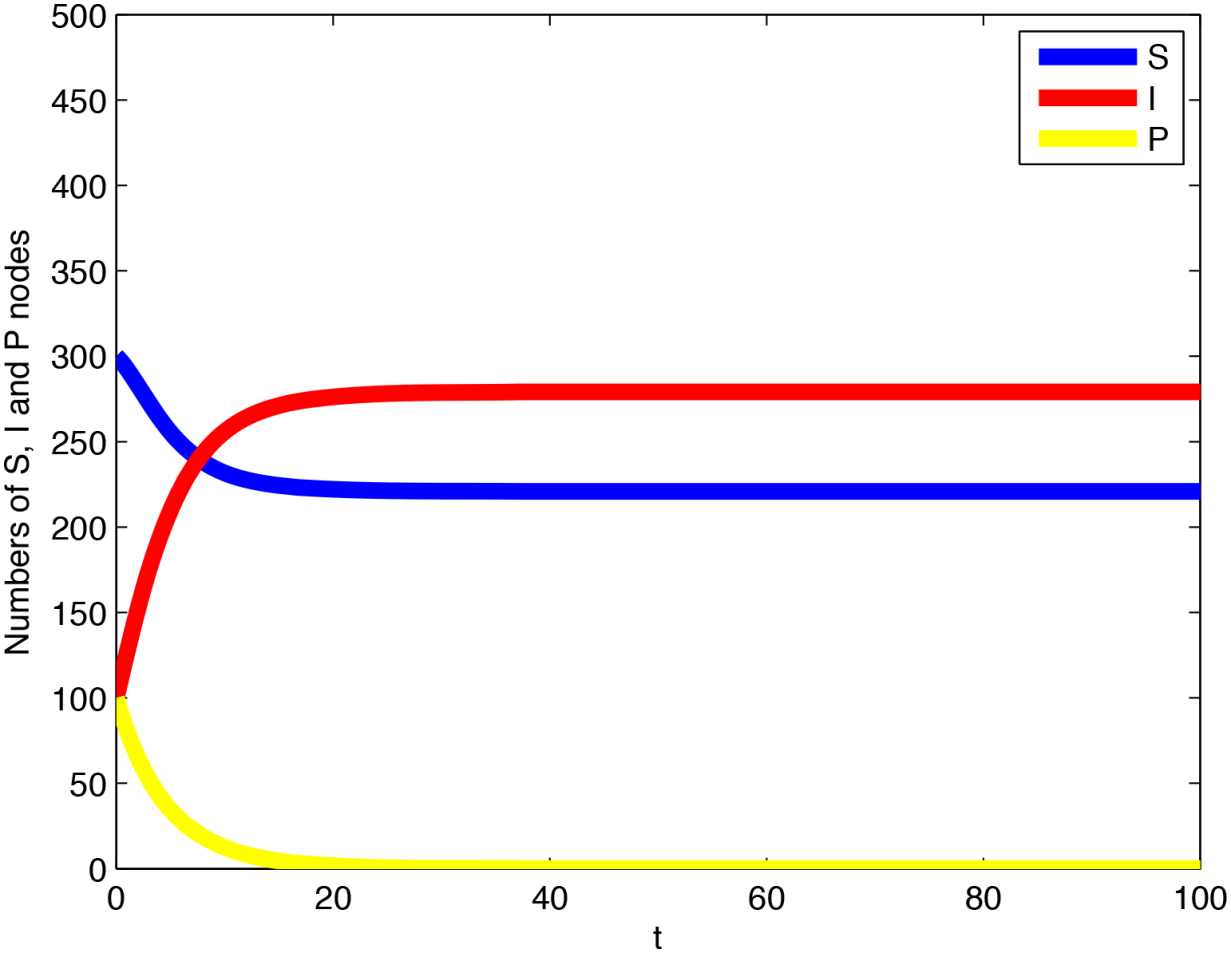
A time plot for the system given in Example 1.

**Fig 4:**
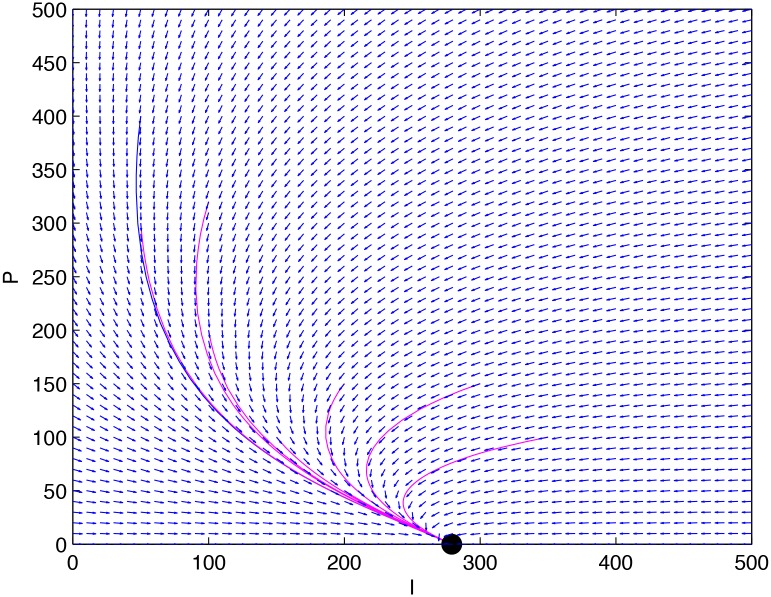
The phase portrait for the equivalent two-dimensional system of the system given in Example 1.

**Example 1**. *Consider system (1) with* *μ* = 100, *δ* = 0.2, *β*_1_ = 0.001, *β*_2_ = 0.1, *γ*_1_ = 0.0002, *γ*_2_ = 0.1, *and* *α* = 0.1. *As* $\frac{\mu }{\delta }<\frac{\alpha +\delta }{\gamma_{1}}$ , *it follows from Theorem 5.2 that* ${\tilde{\text{E}}}_{1}$ *is asymptotically stable with respect to the whole* Ω. Fig 3 *shows a time plot for the system, and* Fig 4 *displays the phase portrait for its equivalent two-dimensional system*.

**Fig 5:**
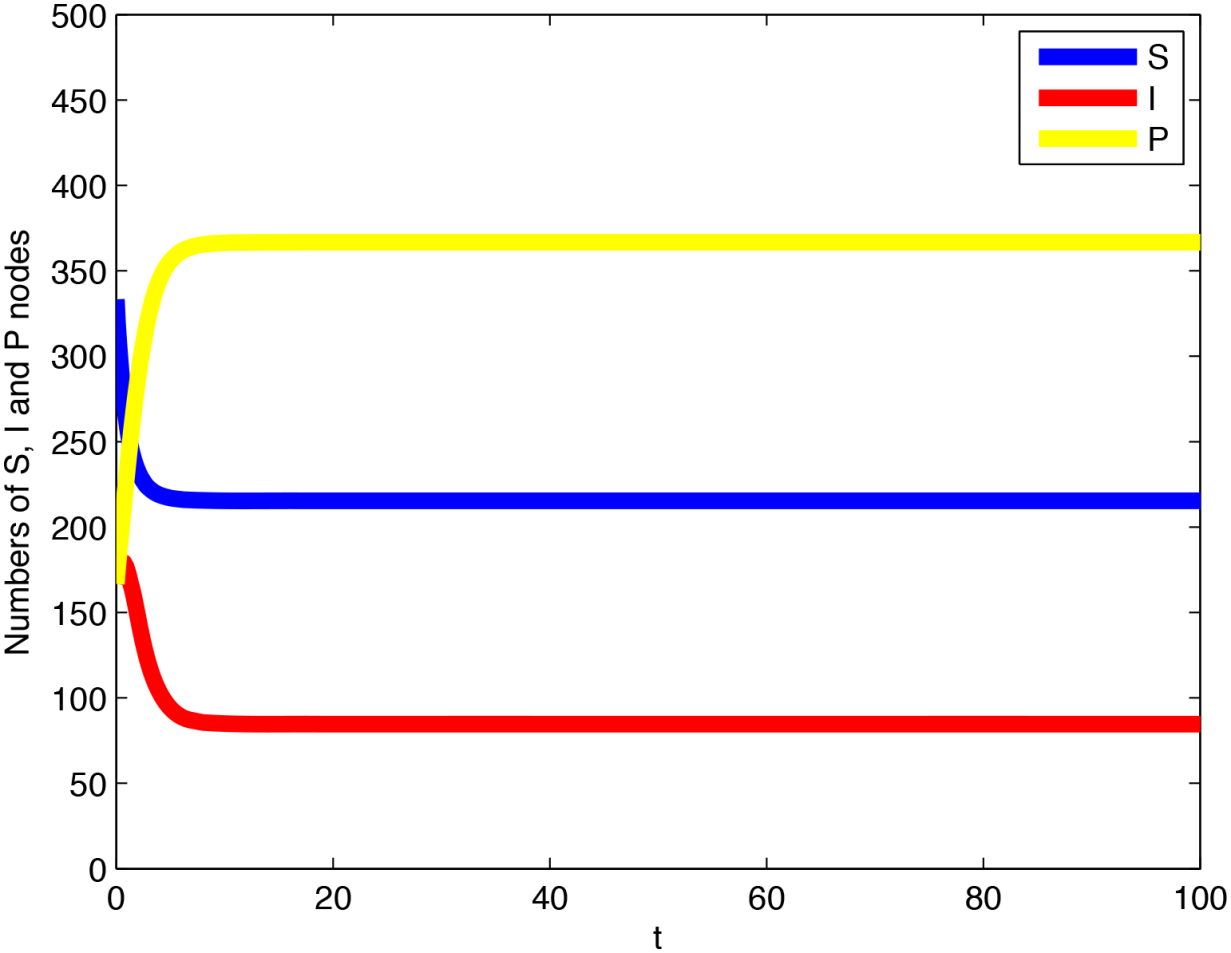
A time plot for the system given in Example 2.

**Fig 6:**
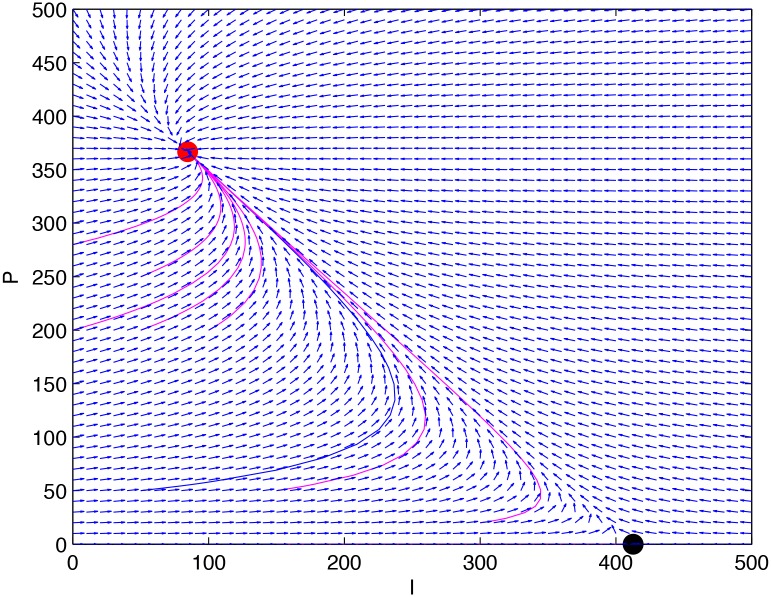
The phase portrait for the equivalent two-dimensional system of the system given in Example 2.

**Example 2**. *Consider system (1) with* *μ* = 200, *δ* = 0.3, *β*_1_ = 0.001, *β*_2_ = 0.4, *γ*_1_ = 0.002, *γ*_2_ = 0.2, *and* *α* = 0.3. *As* $\frac{\mu }{\delta }>\frac{\alpha +\delta }{\gamma_{1}}$ , *it follows from Theorem 5.2 that* ${\tilde{\text{E}}}_{1}$ *is asymptotically stable with respect to* $\Omega -\{(S,I,P)\in R_{+}^{3}:P=0\}$ , *and* ${\tilde{\text{E}}}_{2}$ *is asymptotically stable with respect to* $\Omega -\{(S,I,P)\in R_{+}^{3}:P\neq 0\}$ . Fig 5 *demonstrates a time plot for the system, and* Fig 6 *exhibits the phase portrait for its equivalent two-dimensional system*.

Next, let us use Theorem 5.2 to better suppress electronic viruses. For that purpose, we need the following result.

**Theorem 6.1**. *Consider system (1). Then*, $I_{2}^{*}<I_{1}^{*}$ *if* $\frac{\mu }{\delta }>\frac{\alpha +\delta }{\gamma_{1}}$ .

**Proof**. *Note that*

$$\begin{array}{c} g\left(0\right)=-\beta_{2}\frac{\alpha +\delta }{\gamma_{1}}>-\beta_{2}\frac{\mu }{\delta }=f\left(0\right), \end{array}$$

$$\begin{array}{c} g\left(I_{1}^{*}\right)=\left(\beta_{1}I_{1}^{*}+\beta_{2}+\gamma_{1}I_{1}^{*}\right)\left(\frac{\mu }{\delta }-\frac{\alpha +\delta }{\gamma_{1}}\right)>0=f\left(I_{1}^{*}\right), \end{array}$$

*and* $g(I_{2}^{*})=0$ , *we conclude that* $I_{2}^{*}<I_{1}^{*}$ . *The proof is complete*.

**Fig 7:**
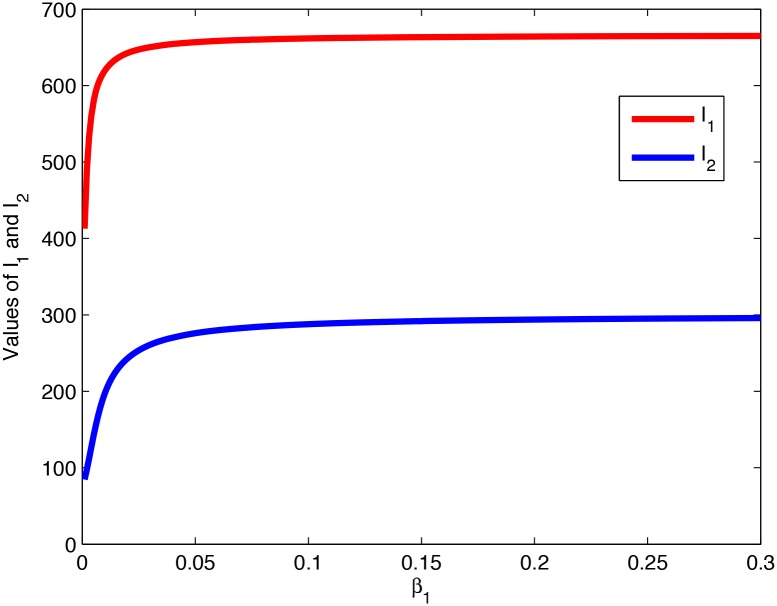
The way *I*_1_ and *I*_2_ vary with the increasing *β*_1_, provided $\frac{\mu }{\delta }>\frac{\alpha +\delta }{\gamma_{1}}$ .

Fig 7 illustrates how $I_{1}^{*}$ and $I_{2}^{*}$ vary with the increasing *β*_1_ provided $\frac{\mu }{\delta }>\frac{\alpha +\delta }{\gamma_{1}}$ , from which it can be seen that $I_{2}^{*}<I_{1}^{*}$ , in agreement with Theorem 6.1.

The parameter $\frac{\mu }{\delta }$ stands for the saturated number of nodes in the Internet and hence is very large. As a result, the condition of $\frac{\mu }{\delta }>\frac{\alpha +\delta }{\gamma_{1}}$ is met in real-world situations. It follows from Theorem 6.1 that a lower $I_{2}^{*}$ value is desired to contain the viral prevalence. A question arises naturally: how can we achieve a lower $I_{2}^{*}$ value? To answer this question, we need the following result.

**Theorem 6.2**. $\frac{\partial I_{2}^{*}}{\partial \delta }>0$ *if* $\frac{\mu }{\delta }>\frac{\alpha +\delta }{\gamma_{1}}$ .

**Proof**. *Differentiating* $I_{2}^{*}$ *with respect to δ on both sides of* Eq (5) *with respect* *δ*, *replace* *I* *with* $I_{2}^{*}$ , *and rearranging the terms, we get*

$$\begin{array}{c} \frac{\partial I_{2}^{*}}{\partial \delta }=\frac{\frac{\beta_{1}}{\gamma_{1}}I_{2}^{*}+\frac{\beta_{2}}{\gamma_{1}}}{2\beta_{1}I_{2}^{*}+\beta_{2}+\gamma_{2}+\frac{\gamma_{1}\mu }{\delta }-\alpha -\frac{\beta_{1}\left(\alpha +\delta \right)}{\gamma_{1}}}>0. \end{array}$$

This theorem shows that reducing the probability per unit time of disconnecting a node from the Internet could benefit the containment of electronic viruses. This interesting phenomenon is attributed to the fact that, compared with the nodes outside the network, the nodes in the network have a chance to acquire the patches for the newest viruses and hence become more robust to malware.

## 7 Conclusions

Assuming that the underlying Internet offers P2P service for every pair of nodes on the network, a dynamic model capturing both the virus propagation mechanism and the distributed patch dissemination mechanism has been proposed. As the infected removable storage media is taken into account, this model captures the real-world situations better than the original SIPS model. The dynamical properties of the proposed model has been fully understood, and it has been found that reducing the probability per unit time of disconnecting a node from the Internet could benefit the containment of electronic viruses.

Towards the evaluation of different distributed patch dissemination strategies, numerous work has yet to be done. First, the proposed model needs modification to adapt to scale-free networks [25, 26] or even general networks [27, 28]. Second, the patch dissemination network may be different from the virus propagation network [2], and future virus-patch dynamical models should characterize this difference. Next, it is worthwhile to study cost-effective patch dissemination strategies by exploiting the optimal control theory [29]. Last, the proposed model can be extended to other situations such as information or rumor propagation [30–32].
